# Lues maligna in a patient living with HIV: A case report

**DOI:** 10.5339/qmj.2025.27

**Published:** 2025-02-05

**Authors:** Jaime Eugenio Espinosa-Mora, Mauricio Alejandro Saldana-Ruiz, Karla Monserrat Ramírez-Pintor, Federico Ortiz-Alonso, Mauricio Linnery Rendón-Saldivar, Luis Alberto Perona-Ramírez

**Affiliations:** ^1^Departamento de Infectología, Unidad Médica de Alta Especialidad No. 25, Instituto Mexicano del Seguro Social, Monterrey, Mexico; ^2^Coordinación Hospitalaria de Donación, Unidad Médica de Alta Especialidad No. 25, Instituto Mexicano del Seguro Social, Monterrey, Mexico; ^3^Facultad de Medicina UANL, Monterrey, Mexico; ^4^Departamento de Medicina Interna, Unidad Médica de Alta Especialidad No. 25, Instituto Mexicano del Seguro Social, Monterrey, Mexico *Correspondence: Mauricio Alejandro Saldana-Ruiz. Email: mauriciomgrr14@gmail.com

**Keywords:** Case report, syphilis, infectious diseases, HIV, lues maligna

## Abstract

**Background:**

Malignant syphilis is a rare form of secondary syphilis that occurs mainly in patients living with human immunodeficiency virus (HIV), with approximately 15 cases reported in the last century. We present the case of a patient treated in our institution.

**Case presentation:**

A 25-year-old male patient presented with round lesions in the form of ulcerations with blackish crust on the plantar area and inner edge of the right foot. VDRL (venereal disease research laboratory) test and ELISA (enzyme-linked immunosorbent assay) were performed for the diagnosis of syphilis and HIV, respectively, which were positive. Subsequently, the patient was hospitalized, and ceftriaxone was indicated due to the lack of crystalline penicillin G in the hospital. Four days later, he had complete improvement of the skin lesions. The patient is currently stable and has no recurrence of skin lesions.

**Discussion:**

Due to the lack of supplies in our unit, we chose ceftriaxone, which is used in patients with penicillin allergies. The use of this drug has shown good outcomes in different reviews.

**Conclusion:**

Due to the use of appropriate treatment, the patient is currently stable and has no recurrence of skin lesions. Malignant syphilis should be considered as part of the differential diagnosis in patients who present with nodulo-ulcerative lesions and have a positive treponemal or non-treponemal test.

## Introduction

Syphilis is a systemic human disease caused by *Treponema pallidum* and is classified as either acquired or congenital. Malignant syphilis is a rare form of acquired syphilis. Formerly known as lues maligna, lues is derived from the Latin word which means “contagious disease”.^1^ First described by Bazin in 1859,^2^ it was initially classified within the spectrum of tertiary syphilis. However, this changed in 1896 when Haslund and Neisser officially classified it as a rare form of secondary syphilis.^3^ It is characterized by papular or nodular lesions with ulceration, no central clearing, and a blackish brown lamellar crust surrounded by an erythematous halo.^4^ We present a case of lues maligna in a patient treated at our tertiary care institution. We received approval from the institutional review board, and informed consent was obtained from the patient.

## Case Presentation

**Figure 1.**
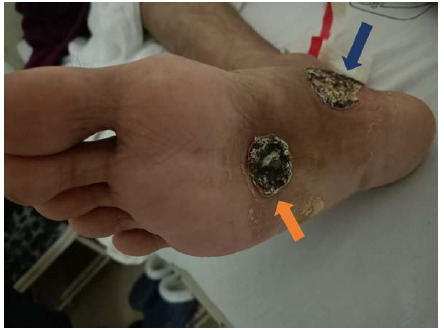
Ulcerated lesion with blackish crust on the plantar surface (orange arrow) and in the Achilles tendon of the right foot (blue arrow) at the time of the patient's initial presentation.

We present the case of a 25-year-old male with a history of retinal detachment of the left eye secondary to panuveitis six months ago, who was treated in our third level of care inpatient unit of the Mexican Institute of Social Security in Mexico. One month later (March 2022), he presented with loss of vision in the same eye and round lesions with ulceration and blackish crust on the plantar area and inner edge of the right foot (Figure 1), which began as vesicles. He also reported unquantified weight loss over two months.

At this time, he was evaluated by the ophthalmology department and diagnosed with persistent left panuveitis. In addition, a VDRL (venereal disease research laboratory) test for syphilis and an ELISA (enzyme-linked immunosorbent assay) test for human immunodeficiency virus (HIV) were positive. Unfortunately, due to a lack of supplies, it was not possible to perform a *Treponema pallidum* particle agglutination test. He was then referred to the department of internal medicine, which administered 2.4 million international units of penicillin G benzathine intramuscularly per week for three weeks and obtained viral load for HIV and CD4 lymphocyte count. The viral load was 145,000 copies/ml and a CD4 lymphocyte count of 4. Treatment was started with bictegravir 50 mg/emtricitabine 200 mg/tenofovir alafenamide 25 mg (Biktarvy) 1 tablet every 24 hours.

He was subsequently hospitalized for suspected ocular syphilis and evaluation of ulcers on the right lower limb. Unfortunately, due to a lack of resources, it was not possible to perform a histopathological examination of the lesion. Then, he had a fever of 38.5°C, so he was given two tablets of trimethoprim–sulfamethoxazole 80/400 mg every 24 hours as prophylaxis for *Pneumocystis jirovecii* and ceftriaxone 2 g intravenously every 24 hours due to the lack of crystalline penicillin G in the hospital. A lumbar puncture was also performed (Table 1).

Four days later, he had complete improvement in the skin lesions and ocular sequelae of visual alteration. However, as he persisted with febrile episodes and non-productive cough, a computed tomography scan was performed, which revealed a cavitary pulmonary lesion.

*Mycobacterium tuberculosis* was diagnosed by polymerase chain reaction, which was treated with isoniazid, rifampicin, pyrazinamide, and ethambutol, in addition to adjusting antiretroviral treatment to dolutegravir 50 mg every 12 hours plus emtricitabine 200 mg/tenofovir disoproxil fumarate 245 mg every 24 hours and follow-up. He complied with the treatment, and had no recurrence of skin lesions and non-productive cough. However, sequelae of decreased visual acuity persisted.

## Discussion

Reported cases of malignant syphilis were extremely rare, with only 14 cases reported between 1900 and 1988.^5^ However, it has increased in the last 30 years.^6^ It occurs more frequently in patients with HIV infection and its pathogenesis is unknown. However, it is believed that immunosuppression due to HIV co-infection allows *Treponema pallidum* to act more actively.^6^

Although rare, case reports and series suggest that HIV patients may have a higher incidence of lues maligna than patients without HIV. It has been found that in patients with HIV, the depletion of CD4 T lymphocytes leads to greater activity of cytotoxic T lymphocytes and neutrophils in the skin, which could help distinguish malignant syphilis more easily from other pathologies.^7^

The currently recommended treatment according to the Centers for Disease Control and Prevention guidelines is based on the administration of penicillin G benzathine for secondary syphilis and aqueous penicillin G for neurosyphilis or ocular syphilis.^8^

This treatment has proven effective in cases of malignant syphilis, as in the study by Fustà-Novell et al.,^9^ which reviewed the cases of four patients diagnosed with malignant syphilis in their hospital unit during 2012–2016. The treatment used in three of their cases was based on the administration of one or three doses of 2.4 MU IM (intramuscular) benzathine penicillin G and the administration of 2.4 MU IM aqueous penicillin G daily for 15 days in the fourth case, which differed due to its presentation of neurosyphilis. All treatment regimens were successful.

The case report by Wang et al. shows the efficacy of penicillin treatment and the classic presentation of the disease in their patient, which is consistent with the presentation of our patient.^10^

Although ceftriaxone-based treatment was chosen due to the shortage of supplies in our unit, this antibiotic showed good results since penicillin G benzathine was previously administered without a positive response. This regimen is used in patients with penicillin allergy, as Wibisono et al. demonstrated in their systematic review.^6^

## Conclusion

Due to the increase in cases of syphilis in people living with HIV, malignant syphilis should be considered as part of the differential diagnosis in patients who present with nodular lesions with ulceration and have a positive treponemal or non-treponemal test.

The outcome of the patient was favorable and there was no recurrence of the lesions two years later, proving that the alternative treatment with ceftriaxone was successful. Therefore, it could be considered a viable therapeutic alternative in some cases.

### Competing interests

The authors have no conflicts of interest to declare.

## Figures and Tables

**Table 1.** Results of lumbar puncture during the patient's hospitalization after the patients’ HIV diagnosis.

	Normal values	Case report of the patient's laboratory results

White blood cells	0–5 cells	3 cells

Glucose	40–70 mg/dL	32 mg/dL

Protein	15–45 mg/dL	142.1 mg/dL

